# An Exploration of Tri-Axial Accelerometers in Assessing the Therapeutic Efficacy of Constraint-Induced Movement Therapy in Children with Unilateral Cerebral Palsy

**DOI:** 10.3390/s23239393

**Published:** 2023-11-24

**Authors:** Youngsub Hwang, Jeong-Yi Kwon, Yoonju Na

**Affiliations:** 1 Department of Health Sciences and Technology, Samsung Advanced Institute for Health Sciences and Technology, Sungkyunkwan University, Seoul 06355, Republic of Korea; asiaargento@naver.com; 2 Department of Physical and Rehabilitation Medicine, Samsung Medical Center, Sungkyunkwan University School of Medicine, Seoul 06351, Republic of Korea; yoonju.na@samsung.com

**Keywords:** tri-axial accelerometers, real-time limb activity monitoring, precision medicine, unilateral cerebral palsy, constraint-induced movement therapy

## Abstract

This study aimed to elucidate the role of tri-axial accelerometers in assessing therapeutic interventions, specifically constraint-induced movement therapy (CIMT), in children with unilateral cerebral palsy (UCP). The primary focus was understanding the correlation between the actigraphy metrics recorded during CIMT sessions and the resultant therapeutic outcomes. Children with UCP, aged between 4 and 12 years, participated in this study from July 2021 to December 2022. In conjunction with in-clinic sessions, during which participants wore tri-axial accelerometers on both limbs, we analyzed actigraphy data over three days of routine activities pre- and post-CIMT. While not all metrics derived from the accelerometers indicated significant improvements post-intervention, there was a clear trend towards a more balanced usage of both limbs, particularly evident in Axis 3, associated with vertical movement (*p* = 0.017). Additionally, a discernible correlation was observed between changes in the magnitude ratio derived from actigraphy data during CIMT (Δweek3–week1) and variations in traditional assessments pre- and post-intervention (ΔT0–T1), specifically the Assisting Hand Assessment grasp and release. Using tri-axial accelerometers has helped clarify the potential impacts of CIMT on children with UCP. The preliminary results suggest a possible link between actigraphy metrics taken during CIMT and the subsequent therapeutic outcomes determined by standardized tests.

## 1. Introduction

Cerebral palsy (CP) is the predominant cause of chronic childhood physical disability in industrialized societies, with prevalence rates of 2–3 per 1000 live births [1]. This neurological condition stems from an injury to the developing brain, primarily manifesting in movement and posture anomalies [2]. Consequently, children diagnosed with CP form the principal cohort within pediatric rehabilitation [3], with their distinct challenge being the hindered progression of gross motor functions [4].

Among the CP spectrum, unilateral CP (UCP)—characterized by motor impairments predominantly on one side—is the most prevalent, representing 30–40% of CP cases [5,6]. For children grappling with UCP, the aberrant movement patterns in their impaired upper limb (UL) directly translate to diminished unimanual capacity and bimanual performance. This subsequently disrupts their ability to perform routine activities [7]. A distinguishing feature of UCP is the phenomenon of ‘developmental disregard’, where the affected side is inadvertently neglected, further deepening the motor function disparity [8].

Clinicians have long relied on various assessment tools and classification systems for evaluating the upper limbs (ULs) in children with UCP. Historically, tools such as the Melbourne Assessment of Unilateral Upper Limb Function (MUUL, later upgraded to MA2) [9] have stood out for their ability to assess unimanual capacity. Others, including the Shriners Hospital Upper Extremity Evaluation (SHUEE) [10] and the Quality of Upper Extremity Skills Test (QUEST) [11], have found their rightful place in evaluations. Notably, the Assisting Hand Assessment (AHA) [12] has gained recognition as a top performance-based measure for bimanual UL activities in these children. At the same time, parent reporting tools, like ABILHAND-Kids [13], have added depth with their nuanced perspectives.

However, with advances in information and communication technology (ICT) and wearable sensors such as three-axis accelerometers, the assessment environment is undergoing a paradigm shift, allowing for a more dynamic approach to assessments [14]. These technologies extend beyond simply assessing motor capacity. They focus on capturing the real-world hand performance of a child, offering continuous and real-time data. By capturing this information in real-world settings, actigraphy can reveal valuable insights into the child’s functional abilities and progress beyond the constraints of controlled environments often associated with standardized assessments [15]. This enhances the ecological validity of the evaluation process, ensuring that the assessment accurately reflects the child’s functional abilities in everyday situations.

Constraint-Induced Movement Therapy (CIMT), one of the foremost intensive therapeutic approaches for UCP, has begun integrating tri-axial accelerometers in its methodology [15,16,17]. This incorporation stems from actigraphy’s ability to offer quantitative insights into limb use, movement symmetry, and general activity. Researchers have been keen on capturing parameter changes like use ratio, magnitude ratio, and bilateral magnitude [15,16,17]. However, despite actigraphy’s potential to enhance CIMT research, its integration remains in its early stages. A prevailing challenge is the discernible absence of standardization among studies, leading to data consistency and comparability questions. If such variability remains to be addressed, it may overshadow the progress and innovative breakthroughs awaiting discovery in this domain.

In this study, we sought to elucidate the role of tri-axial accelerometers in the therapeutic context of UCP. Our primary objective was to determine how much actigraphy metrics recorded during CIMT sessions correlate with therapeutic outcomes. Furthermore, we rigorously examined the differential behavioral patterns of the affected and less-affected extremities throughout CIMT. Moreover, by comparing actigraphy variables before and after CIMT, we aim to offer a systematic insight into the quantifiable changes attributable to the therapy.

## 2. Materials and Methods

### 2.1. Study Design and Population

This single-arm trial (Registration number: NCT04904796), conducted between July 2021 and December 2022, targeted children aged 4 to 12 with UCP. Participants were recruited from the outpatient department of a tertiary hospital in Seoul, the Republic of Korea. Eligible children had UCP diagnoses from central nervous system lesions, whereas those with severe cognitive dysfunction, untreated seizures, vision or hearing impairments, or a history of musculoskeletal disorders were excluded. The hospital’s review board approved the study (Approval number: SMC 2021-04-042), and parents or legal guardians provided informed consent before enrollment.

### 2.2. Procedure

**Figure 1:**
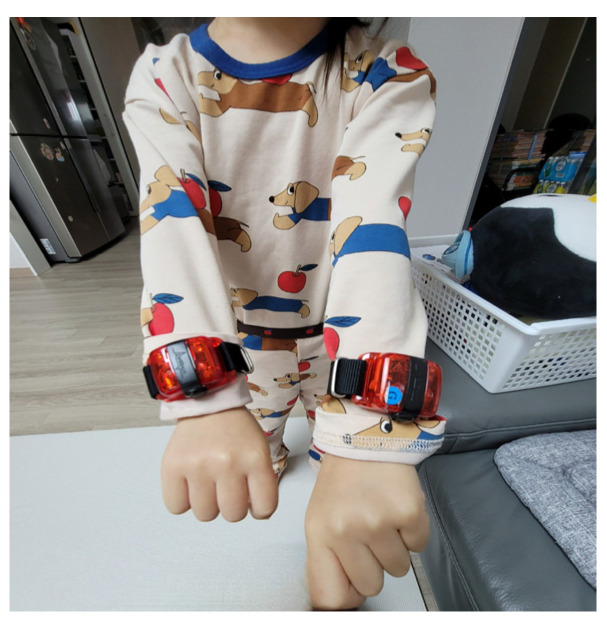
A child wearing the accelerometer in daily life settings (at T0 and T1). A child participant wearing accelerometers during daily activities. This image illustrates the practical application of the ActiGraph wGT3X-BT accelerometers in a real-life setting, as part of the data collection process at baseline (T0) and at the post-intervention time point (T1).

**Figure 2:**
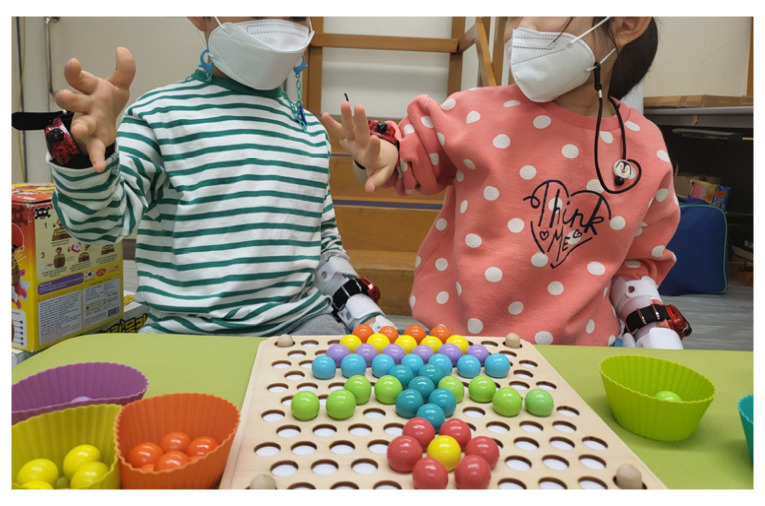
Children wearing the accelerometer during CIMT sessions in the clinic. This image showcases the application of accelerometry in a controlled environment, highlighting the in-clinic phase of the study.

During the study, children wore accelerometers on both wrists for three consecutive days at two different time points: baseline (T0) and the post-intervention time point (T1) (Figure 1). Within one week after the accelerometry evaluation, standardized assessments were administered to evaluate the children’s progress. Standardized assessments were conducted by an occupational therapist with over 10 years of experience and performed single-blindly. At the intervention phase (between T0 and T1), the participants underwent three weeks of CIMT (15 sessions, 5 sessions per week, 2 h per session). Notably, during these CIMT sessions in a clinical setting, children wore accelerometers on both hands throughout the duration (Figure 2).

### 2.3. Physical Activity Monitoring Using Accelerometer-Based Monitors

Activity data was acquired using the ActiGraph wGT3X-BT, a compact (4.6 × 3.3 × 1.5 cm) tri-axial accelerometer. This wireless device records acceleration across three planes, with a dynamic range of up to ±8 gravitational units at 30 Hz. For our study, these devices were securely attached to the children’s wrists on the affected and less-impacted sides. Of the metrics derived from accelerometers, we prioritized the Vector Magnitude Average counts (VMA) and the VMA ratio. The VMA represents the cumulative activity count spanning three distinct axes of movement. Specifically, Axis 1 measures the forward and backward motions of the hand, Axis 2 captures side-to-side movements, and Axis 3 denotes vertical up-and-down movements. The VMA ratio, on the other hand, is the natural logarithm of the ratio of the affected side’s VMA to the less-affected side’s VMA. The data captured was initially processed in 10 s intervals using ActiLife 6 software (ActiGraph, Pensacola, FL, USA) and then translated into activity counts.

### 2.4. In-Laboratory Standardized Assessments

The Pediatric Evaluation of Disability Inventory Computer Adaptive Test (PEDI-CAT) [18] is a recognized tool gauging functional capabilities in young people (from birth to 20 years) across different health conditions. It relies on a 276-item adaptive system based on caregiver insights, covering four areas: movement, day-to-day tasks, social/cognitive abilities, and autonomy. Each area is scored from 20 (minimal function, high caregiver involvement) to 80 (optimal function, minimal caregiver involvement). Although two versions exist, we opted for the Speedy variant due to its streamlined process.

The Pediatric Motor Activity Log (PMAL) [19] tracks the application of a child’s predominantly impacted UL during everyday actions. It features 22 arm–hand tasks, with data being systematically categorized into (1) frequency (HO) and (2) proficiency (HW).

The Melbourne Assessment 2 (MA2) [20] is a specific UL assessment focusing on the capacity of one hand. It benchmarks against specific criteria, delving into movement range, accuracy, agility, and flow. The test was videotaped for analysis, and the raw score was converted to a percentage of the maximal score.

The Assisting Hand Assessment (AHA) [21] evaluates a child’s propensity to use their impaired hand in tandem with the other during bimanual tasks. The 22-item AHA session was videotaped and rated on a 4-point scale. The raw scores were converted to interval-level data using a Rasch analysis, providing linearly rescaled Rasch measurements ranging from 0 to 100 AHA units. Higher numbers indicate higher ability.

The Canadian Occupational Performance Measure (COPM) [22] was designed to identify and measure, using interviews, changes in functional problems clients consider relevant to self-care, productivity, and leisure activity performance. The client or caregiver defines the most relevant functional goals to be accomplished, ranks their importance, and rates the child’s performance ability and satisfaction level.

### 2.5. Statistical Analyses

Descriptive statistics were used to summarize the demographic and clinical characteristics of the participants. A paired *t*-test was performed to compare pre- and post-intervention differences in standardized assessments and actigraphy variables. For a nuanced examination of actigraphy measurements, we segmented the data accumulated during the 3-week CIMT sessions into weekly averages: Week 1 reflected the mean values derived from sessions 1–5, Week 2 from sessions 6–10, and Week 3 from sessions 11–15. Our focal point of the correlation analysis was the relative change in these weekly actigraphy averages across the CIMT duration, calculated as (Value at Week 3–Value at Week 1)/Value at Week 1 × 100. This provided a comprehensive view of the progression over the three weeks. Using the Pearson correlation analysis, we then investigated how this rate of change correlated with differences observed in the metrics before and after the entire intervention. We considered *p* = 0.05 as the cut-off for statistical significance and deemed Pearson’s correlation coefficients ±0.5 to represent a strong correlation. All statistical analyses were performed using SAS 9.4 (SAS Institute Inc., Cary, NC, USA) and R 3.5.0 (R Foundation for Statistical Computing, Vienna, Austria).

## 3. Results

**Figure 3:**
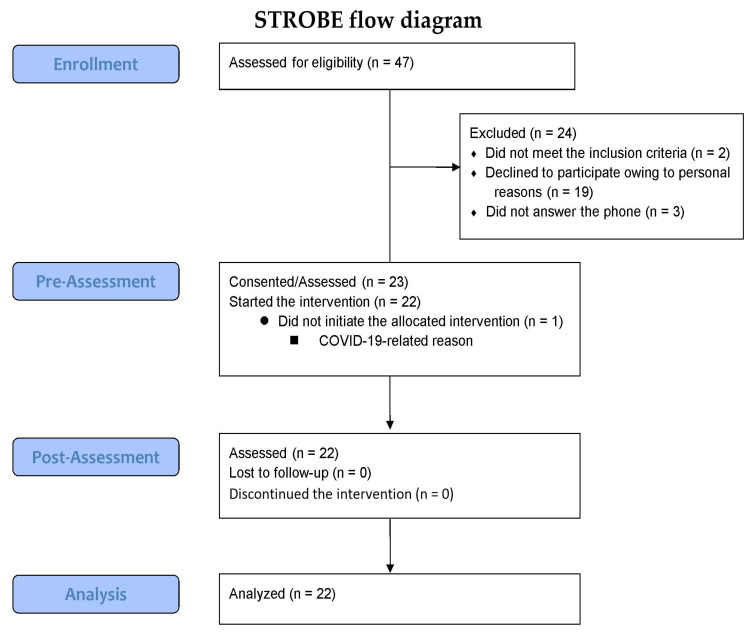
Study enrollment flow chart.

A total of 47 participants were assessed for eligibility, of which 24 were excluded due to not meeting the inclusion criteria (n = 2), declining to participate due to personal reasons (n = 19), or not answering the phone (n = 3). The remaining 23 participants consented and underwent the pre-assessment, but one participant did not start the allocated intervention due to concerns related to COVID-19. Consequently, 22 participants completed the three-week intervention and subsequent post-assessment, and their data were included in the final analysis (Figure 3). The characteristics of the children are presented in Table 1 and Supplementary Table S1.

Table 1 presents the demographic and clinical breakdown of the participants. The average age of the participants was 5.48 years (SD = 1.85). Of the participants, 54.54% (n = 12) were boys, and 45.46% (n = 10) were girls. Regarding the MACS level, 31.83% (n = 7) were at level 1, 45.45% (n = 10) at level 2, and 22.72% (n = 5) at level 3. The majority, 77.27% (n = 17), had their right side involved, while 22.73% (n = 5) had the left side.

### 3.1. Correlation Analysis of Actigraphy Changes over the CIMT Sessions (ΔWeek3–Week1) with Pre-Post Differences in the Standardized Assessments (ΔT1–T0)

**Figure 4:**
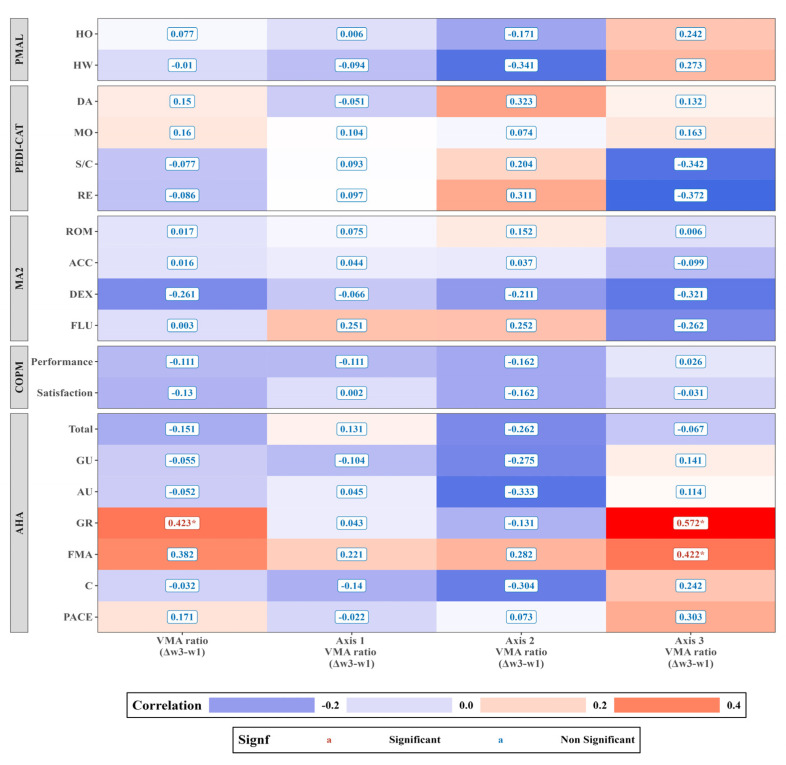
Correlations between changes in the standardized assessments (ΔT0–T1) and actigraphy variations throughout the CIMT sessions (Δweek3–week1) in a clinical environment. The heatmap vividly captures the correlation analysis between changes in the standardized assessment results from pre- to post-CIMT (ΔT0–T1) and actigraphy variations throughout the CIMT sessions (Δweek3–week1). In the visualization, the warmer tones (reds) represent positive correlations, and the cooler tones (blues) represent negative correlations between the actigraphy variations and the improvements in the standardized assessment scores. * *p* < 0.05; *y*-axis abbreviations: HO, how often; HW, how well; DA, daily activities; MO, mobility; S/C, social/cognitive; RE, responsibility; ROM, range of motion; ACC, accuracy; DEX, dexterity; FLU, fluency; GU, general use; AU, arm use; GR, grasp and release; FMA, fine motor adjustment; C, coordination.

Figure 4 presents the correlation analysis changes in the standardized assessment outcomes before and after the intervention (ΔT0–T1) and the changes in actigraphy metrics observed throughout the CIMT sessions (Δweek3–week1). A significant association was evident between the fluctuation in the VMA ratio (Δweek3–week1) across the CIMT sessions and the alterations in AHA grasp and release (GR) from pre- to post-intervention (ΔT1–T0), showcasing a Pearson correlation coefficient of 0.423 (*p* = 0.049). Notably, Axis 3 VMA ratio’s change (Δweek3–week1) across the sessions exhibited a robust correlation with the change in AHA GR (ΔT1–T0), reflecting a Pearson correlation coefficient of 0.572 (*p* = 0.006). Additionally, the Axis 3 VMA ratio (Δweek3–week1) was significantly correlated with the AHA fine motor adjustment (FMA) change from pre- to post-intervention (ΔT1–T0), registering a Pearson correlation coefficient of 0.422 (*p* = 0.049).

### 3.2. Correlations of Daily Life Actigraphy Alterations (ΔT1–T0) with Changes Observed over the CIMT Sessions (ΔWeek3–Week1) in a Clinical Setting

**Figure 5:**
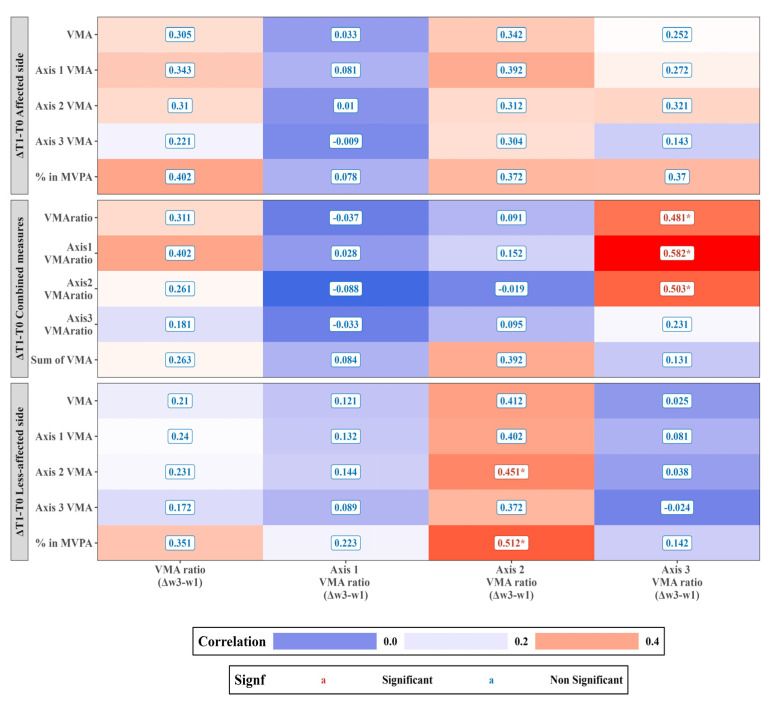
Correlations between actigraphy changes recorded over three days (ΔT0–T1) in daily life settings and actigraphy variations throughout the CIMT sessions (Δweek3–week1) in a clinical environment. Heatmap of the relationship between the change in actigraphy variables during CIMT sessions in a clinical environment (Δweek3–week1) (*x*-axis) and the difference in the actigraphy data before and after the intervention in daily life settings (ΔT0–T1) (*y*-axis). In the visualization, the warmer tones (reds) represent a positive correlation, and the cooler tones (blues) represent negative correlations between the use of the paretic side during CIMT sessions (as evidenced by higher Δweek3–week1 values) and actigraphy data changes in daily life (larger ΔT0–T1 values). * *p* < 0.05.

Figure 5 presents the correlation analysis between actigraphy changes recorded over three consecutive days (ΔT0–T1) in daily life settings and changes over the CIMT sessions (Δweek3–week1) in a clinical setting. Notably, the alterations in the Axis 2 VMA ratio (Δweek3–week1) during the CIMT sessions significantly correlated with two actigraphy metrics: Axis 2 VMA of the less-affected side (ΔT1–T0) and the % in MVPA (moderate-to-vigorous physical activity) of the less-affected side (ΔT1–T0), showcasing correlation coefficients of 0.451 (*p* = 0.035) and 0.512 (*p* = 0.015), respectively. This Axis 2 VMA ratio (Δweek3–week1) was also positively associated with VMA metrics such as the less-affected side VMA, Axis 1 VMA, and Axis 3 VMA.

The Axis 3 VMA ratio (Δweek3–week1) showed significant correlations with the VMA ratio (ΔT1–T0), Axis 1 VMA ratio (ΔT1–T0), and Axis 2 VMA ratio (ΔT1–T0), with respective correlation coefficients of 0.481 (*p* = 0.024), 0.582 (*p* = 0.005), and 0.503 (*p* = 0.018). The VMA ratio (Δweek3–week1) and Axis 2 VMA ratio (Δweek3–week1) showed a positive correlation trend with the Axis 1 VMA ratio (ΔT1–T0) and Sum of VMA (ΔT1–T0), respectively.

### 3.3. Comparison of the Vector Magnitude Average Counts (VMA) between the Baseline (T0) and Post-Intervention Time Point (T1)

Table 2 displays the VMA comparisons between the initial assessment (T0) and post-intervention time point (T1) for both the affected and less-affected sides. On the affected side, the VMAs of all axes increased from 463.95 ± 145.56 at T0 to 497.35 ± 156.20 at T1, though this rise was not statistically significant (*p* = 0.152). Similar nonsignificant increments were seen for the three individual axes. For the less-affected side, slight increases were observed across all VMAs between T0 and T1, but none reached statistical significance. Regarding the combined measures, including the VMA ratios for each axis, only marginal variations were seen between the two time points. Notably, the VMA ratio for Axis 3 showed a change from −0.60 ± 0.29 at T0 to −0.53 ± 0.29 at T1 (*p* = 0.017). This change in the VMA ratio suggests a trend toward a more balanced use of both limbs, moving closer to zero, which represents the equal use of both sides. The VMAs for both sides also increased from 1236.61 ± 313.89 at T0 to 1294.84 ± 323.21 at T1 without reaching statistical significance (*p* = 0.236).

### 3.4. Weekly Actigraphy Trends across the Three-Week CIMT Duration

Table 3 presents the fluctuations in actigraphy outcomes observed during the three-week course of CIMT. For the less-affected side, there was a general decline in the VMA values across the weeks, with the VMAs of all axes reducing from 238.42 ± 148.54 in the first week to 213.24 ± 125.28 by the third week. Similar descending trends were observed for individual axes, with VMA Axis 1, Axis 2, and Axis 3 all showing a decrease over the three weeks. In contrast, on the affected side, the VMAs seemed to increase slightly, although not consistently, across the weeks. The VMAs for all axes increased from 552.35 ± 152.85 in the first week to 568.23 ± 153.07 in the second week, with a slight dip to 565.67 ± 173.68 in the third week. Similarly, the VMAs for Axis 1 and Axis 2 saw an upward trend, while Axis 3 remained relatively consistent across the weeks. When examining the ratios, the VMA ratio increased over the three weeks, from 0.99 ± 0.54 in the first week to 1.11 ± 0.56 in the third week. A similar pattern was observed for the Axis 1, Axis 2, and Axis 3 ratios, indicating a progressive balancing between the affected and less-affected sides during the CIMT period (Supplementary Figure S1).

## 4. Discussion

Our investigation focused on the VMA ratio—a biomarker that quantifies the movement disparity between the affected and less-affected upper extremities in children with UCP. The VMA ratio, calculated as the VMA of the affected side divided by the VMA of the less-affected side, has been increasingly recognized as an informative measure in UCP research [15,16,17]. The distinctive feature of our study is rooted in its innovative methodology. We equipped both the restrained, less-affected and affected limbs with accelerometers, ensuring consistent and comprehensive monitoring of the VMA ratio throughout the CIMT sessions. Traditionally, emphasis on the VMA ratio has predominantly centered on pre- and post-therapy evaluations, rather than capturing its dynamic fluctuations during the therapy sessions. The VMA ratio’s ability to capture changes in mirror movements, a substantial determinant of therapeutic outcomes [23], underscores the need for its evaluation based on data collected during therapy sessions. Previous research, including the efforts of Coker-Bolt et al. [16], initiated the integration of accelerometer-based monitoring during CIMT sessions. However, their approach fell short by excluding the restrained side, resulting in a missed opportunity to capture crucial changes in the VMA ratio. Similarly, Goodwin et al. [17] managed to equip both limbs with accelerometers; however, their approach oversimplified the analysis by averaging data across the therapy’s entire duration, thereby neglecting the intricacies of weekly limb movement dynamics. In contrast, our study addresses these shortcomings, providing a more nuanced understanding of movement variations during CIMT sessions for children with UCP. This advancement represents a significant stride in our approach to evaluating and comprehending the effects of therapeutic interventions in this specific domain.

The current study explored the potential correlation between actigraphy changes recorded throughout CIMT sessions (Δweek3–week1) and pre-post CIMT standardized assessment outcomes in children with UCP. We noticed an alignment between in-session actigraphy variations and post-therapy standardized assessments. Our detailed analysis showed that the VMA ratio (Δweek3–week1) changes correlated with changes in the AHA GR (ΔT1–T0). Furthermore, our findings revealed a noteworthy association between the VMA ratio derived from Axis 3 and the AHA GR and FMA. This underscores the significant usefulness of monitoring within a clinical setting for predicting therapeutic outcomes. Notably, the VMA ratio (Axis 2) captured during CIMT sessions correlated with the VMA ratio derived from three-day real-world measurements conducted pre- and post-therapy. This alignment emphasizes how tri-axial accelerometers can effectively measure the impact of CIMT, bridging in-clinic measurements with patients’ everyday activities. Our results may highlight the role of in-session limb activity monitoring and traditional assessments to fine-tune CIMT protocols, catering better to individual needs. This integration of concurrent monitoring with established therapeutic practices underscores the potential enhancements in UCP rehabilitation methodologies, with considerations for emerging fields such as telerehabilitation [24].

Conversely, the lack of corresponding correlations with assessments such as MA2, PEDI-CAT, COPM, and PMAL may indicate that their measurements consist of different concepts. The MA2 predominantly evaluates specific upper limb function capacity in a context-independent manner, whereas the PEDI-CAT captures a broader spectrum of capabilities within daily life activities. In contrast, the COPM and PMAL consist of subjective evaluations, reflecting personal and caregiver perspectives, which might not correlate with the actigraphy’s objective activity metrics. This distinction highlights the intricate nature of its functional application in the daily lives of children with unilateral cerebral palsy. Actigraphy measures physical activity quantitatively; however, it cannot accurately reflect the nuanced quality of the motor functions, nor the subtleties of the adaptive behaviors post-intervention. Hence, these observations might emphasize the need for an integrated, multidimensional approach to evaluate pediatric rehabilitation. This approach should synergize the objective activity measurements with in-depth assessments of function, thereby providing a more holistic view of a child’s recovery and adaptive progress.

Our findings echo previous research patterns that focused on pre- and post-CIMT changes. Hwang et al. [15] identified an upward trajectory in the VMA ratio post-CIMT, though it did not reach statistical significance. Coker-Bolt et al. [16] observed post-CIMT VMA ratio increases in 5 out of 12 participants. In our cohort, the VMA ratio post-CIMT increased for 10 of the 22 participants, which is consistent with the findings of Coker-Bolt et al. Such consistency suggests that, even with variations in the study settings, intervention content, and population characteristics, there may be underlying therapeutic trends within the UCP population. Various factors, from age, general health, concentration, and motivation to the environmental milieu, may influence these outcomes. Recognizing and quantifying these variables require a comprehensive approach. Future studies should consider regression analyses with larger sample sizes to elucidate these determinants more clearly.

Our study explores the specificities of axes Axis 1, Axis 2, and Axis 3, expanding on the current discourse. Axis 1 captures the anterior–posterior motions, Axis 2 encompasses medial–lateral movements, and Axis 3, which notably demonstrated significant alterations, records vertical movements. The significant alteration observed in Axis 3 was of particular interest. This emphasis on Axis 3 is supported by marked deviations in trunk kinematics observed in children with UCP, especially during forward-reaching tasks, which play an essential role in vertical stabilization [25]. The shoulder’s kinematics, particularly its initial frontal orientation during specific reaching tasks and its diminished endpoint elevation, further reinforces the importance of Axis 3 in capturing the intricacies of vertical movements [25]. We postulated that these kinematic disparities could stem from underlying factors, including muscle tone imbalances or distinct muscle spasticity patterns. Such factors may present inherent challenges for children with upper limb cerebral palsy (UCP) and, consequently, hold the promise of substantial improvement following therapeutic interventions.

Given that vertical movements are fundamental to many daily activities, the therapy might have been particularly attuned or effective in this dimension. The change from −0.60 ± 0.29 at T0 to −0.53 ± 0.29 at T1 for Axis 3 (*p* = 0.017) suggests the potential of tri-axial accelerometers in tracking such variations. This result highlights the potential value of an axis-specific approach in upcoming research. Subsequent research endeavors stand to gain from conducting a more comprehensive examination of these findings. This invites researchers to undertake meticulous and advanced refinements to their measurement methodologies, consequently advancing our comprehension of the intricate interplay between movement patterns and the outcomes of therapeutic interventions.

During the three-week CIMT program, variations in the actigraphy outcomes were observed. VMAs on the less-affected side appeared to decrease steadily, hinting at a potential shift towards using the affected limb. Meanwhile, the affected side showed modest increases, most notably between the first and second weeks. This pattern seems to align with findings from prior research on young children with CP [26], suggesting that CIMT might influence corticospinal tract (CST) reorganization. This indicates the young brain’s adaptive capacity to modify its CST attributes in the presence of interventions such as CIMT. Such observations support the notion that changes in neural activity during CIMT could be achieved without invasive measures, especially in influencing the CST properties in young children with UCP. Additionally, the observation of stabilization or minor declines in some axes between the second and third weeks prompts a reevaluation of the optimal duration of CIMT, suggesting that a shorter, more intensive period might be just as, if not more, effective in eliciting meaningful changes in motor function and neural organization.

This study equipped both limbs of children with UCP with accelerometers during CIMT sessions, enabling a nuanced analysis of mirror movement patterns through comprehensive monitoring of the VMA ratio. The established correlation between in-session actigraphy changes and standardized assessments enhanced our understanding of CIMT’s effectiveness. The axis-specific analysis, particularly regarding vertical movements, further enriched the insight into movement variations during therapeutic interventions. Despite these strengths, this study has its limitations. The small sample size and lack of a control group constrain the generalizability of our results. We did not recruit typically developing children for actigraphy comparison, limiting our understanding of differential movement patterns. We could not filter out ’sway’ data from walking during actigraphy measurements, which could be addressed in future studies with human activity recognition techniques [27]. The heterogeneous nature of our participants introduced potential variability in the treatment responses. Our short follow-up only assessed immediate post-CIMT outcomes, leaving long-term results unknown. Addressing these limitations in future studies will strengthen the evidence base for CIMT’s effectiveness in children with UCP.

## 5. Conclusions

In this study, we explored the utility of tri-axial accelerometers in CIMT for children with UCP. The data suggest a potential correlation between real-time actigraphy metrics during CIMT and subsequent therapeutic outcomes, as evidenced by standardized assessments. Notably, variations in the VMA ratio, especially concerning Axis 3 tied to vertical movements, offer valuable insights that could guide the refinement of therapeutic interventions. This study makes a novel contribution to the literature, as it serves as an initial step in this area of research. It highlights promising areas for future investigation, especially in the nuanced use of tri-axial accelerometers for detailed movement analyses. However, the study’s limitations, including a small sample size and the absence of a control group, call for a cautious interpretation of our findings. Larger-scale studies with a more diverse participant pool are necessary to validate and build upon these initial observations. Such studies would enhance the robustness of the conclusions and contribute significantly to the understanding and effectiveness of CIMT in children with UCP. Future studies should not only aim to corroborate these findings with larger populations and longer-term follow-up periods but also assess the integration of telerehabilitation tools to enhance the delivery and sustainability of CIMT. Embracing telerehabilitation could significantly broaden treatment access and ensure ongoing therapy adherence, enabling more tailored and dynamic rehabilitation strategies. This approach would not only extend the reach of CIMT but also hold the potential to transform the practice by embedding interventions more seamlessly into the daily lives of children with UCP, irrespective of their physical location.

## Figures and Tables

**Table 1:** Demographic and clinical characteristics of the children.

Characteristics		
Age, mean (SD), years		5.48 (1.85)
Sex, n (%)	Male	12 (54.54)
	Female	10 (45.46)
MACS level n (%)	1	7 (31.83)
	2	10 (45.45)
	3	5 (22.72)
Side of involvement, n (%)	Right	17 (77.27)
	Left	5 (22.73)

MACS, Manual Ability Classification System.

**Table 2:** Paired *t*-test outcomes for the T0–T1 actigraphy measures (n = 22).

	T0	T1	*p*
Affected side			
VMA of all axes	463.95 ± 145.56	497.35 ± 156.20	0.152
VMA of Axis1	261.80 ± 83.66	278.74 ± 84.77	0.233
VMA of Axis2	246.79 ± 78.65	265.41 ± 85.36	0.134
VMA of Axis3	258.18 ± 88.12	279.58 ± 98.50	0.125
**Less-affected side**			
VMA of all axes	772.66 ± 182.07	797.50 ± 185.74	0.367
VMA of Axis1	421.20 ± 114.43	439.62 ± 112.14	0.279
VMA of Axis2	407.42 ± 103.81	424.33 ± 104.72	0.251
VMA of Axis3	449.98 ± 98.91	456.76 ± 107.25	0.681
**Combined measures**			
VMA Ratio	−0.54 ± 0.21	−0.50 ± 0.23	0.09
VMA Ratio Axis1	−0.49 ± 0.17	−0.48 ± 0.19	0.349
VMA Ratio Axis2	−0.53 ± 0.18	−0.50 ± 0.22	0.28
VMA Ratio Axis3	−0.60 ± 0.29	−0.53 ± 0.29	0.017 *
Sum of VMA	1236.61 ± 313.89	1294.84 ± 323.21	0.236

Abbreviations: VMA, vector magnitude average counts; Sum of VMA, sum of both sides of the VMA. * *p* < 0.05.

**Table 3:** Weekly changes in the actigraphy measures over the 3-week CIMT.

		Week 1	Week 2	Week 3
**Less-affected side**	VMA	238.42 ± 148.54	222.79 ± 129.6	213.24 ± 125.28
	VMA Axis1	106.69 ± 75.67	94.21 ± 61.39	85.11 ± 56.33
	VMA Axis2	132.70 ± 77.61	126.15 ± 69.64	120.6 ± 67.8
	VMA Axis3	137.53 ± 96.94	127.77 ± 85.55	125.22 ± 83.99
**Affected side**	VMA	552.35 ± 152.85	568.23 ± 153.07	565.67 ± 173.68
	VMA Axis1	310.35 ± 92.3	320.89 ± 90.28	317.26 ± 94.3
	VMA Axis2	321.03 ± 92.05	330.82 ± 100.78	330.25 ± 108.67
	VMA Axis3	269.81 ± 94.12	274.69 ± 94.82	275.63 ± 111.01
**Ratio**	VMA Ratio	0.99 ± 0.54	1.08 ± 0.56	1.11 ± 0.56
	Axis1	1.25 ± 0.57	1.39 ± 0.58	1.48 ± 0.57
	Axis2	1.01 ± 0.56	1.09 ± 0.58	1.15 ± 0.63
	Axis3	0.85 ± 0.65	0.93 ± 0.68	0.92 ± 0.66

Abbreviation: VMA, vector magnitude average counts.

## Data Availability

The data are available on request due to privacy restrictions.

## Supplementary Materials

The following supporting information can be downloaded at https://www.mdpi.com/article/10.3390/s23239393/s1: Table S1: Participant characteristics and neuroimaging findings. Figure S1: Weekly trends in actigraphy outcomes during CIMT.
