# Supplementary material for: An Exploration of Tri-Axial Accelerometers in Assessing the Therapeutic Efficacy of Constraint-Induced Movement Therapy in Children with Unilateral Cerebral Palsy
Source: Sensors (Basel). 2023 Nov 24;23(23):9393. doi: 10.3390/s23239393 (PMC10708848; doi:10.3390/s23239393)
Supplement: Supplementary file 1 [file sensors-23-09393-s001.zip › sensors-2707139-supplementary.pdf]

Supplementary Material

Article

An Exploration of Tri-axial Accelerometers in Assessing the Therapeutic Efficacy of Constraint-Induced Movement Therapy in Children with Unilateral Cerebral Palsy

Youngsub Hwang [1], Jeong-Yi Kwon [2]* and Yoonju Na [2]

[1] Department of Health Sciences and Technology, Samsung Advanced Institute for Health Sciences and Technology, Sungkyunkwan University, Seoul, Republic of Korea

[2] Department of Physical and Rehabilitation Medicine, Sungkyunkwan University School of Medicine, Samsung Medical Center, Seoul, Republic of Korea

*Correspondence: Jeong-Yi Kwon

Email: jeongyi.kwon@samsung.com

Table of contents

Supplementary Table S1. Participant characteristics and neuroimaging findings

Supplementary Figure S1. Weekly Trends in Actigraphy Outcomes During CIMT

Supplementary Table S1. Participant characteristics and neuroimaging findings

Child	Sex	Corrected age (y)	Birth term	Basic pattern of damage	Affected hemisphere	Cortical lesion	Involvement of central nuclei	FSIQ
1	M	5.17	Full	MCA infarction	L	F, P	BG	111.00
2	F	5.00	Pre	MCA infarction	L	F, P, T	BG, TH	73.00
3	M	5.42	Pre	N/A				103.00
4	M	6.50	Pre	MCA hemorrhagic infarction	L	F, P	BG, TH	52.00
5	F	5.00	Full	Dysplastic cortex and brainstem, WMDI	L	F, P, T	TH	109.00
6	F	4.42	Pre	HIE	L>R	T, P, O	CR	51.00
7	F	9.58	Full	MCA hemorrhagic infarction	L	F, P, T	BG, TH	64.00
8	M	4.33	Pre	Hemorrhage	R	F, P	TH, BG, PO	84.00
9	M	4.08	Full	N/A				117.00
10	M	10.58	Pre	N/A				70.00
11	F	4.00	Pre	WMDI & hemorrhagic infarction	R	F, P, T	BG, TH	N/A
12	M	9.17	Pre	IVH	L	P, T	BG, TH	75.00
13	M	4.00	Full	MCA infarction	L	F, P	TH	72.00
14	M	4.17	Full	HIE	L	F, P	BG, TH	100.00
15	F	4.50	Full	HIE	L	F, P, T	TH	86.00
16	F	5.08	Full	HIE	R	F	TH, CR	105.00
17	F	5.83	Pre	IVH	L	F, P, O	TH	73.00
18	M	4.33	Pre	WMDI	R, L	F	-	123.00
19	M	6.00	Full	MCA hemorrhagic infarction	L	F	BG, TH	44.00
20	M	4.00	Full	MCA infarction	R	F, P	BG, TH	103.00
21	F	5.42	Pre	HIE	R	F, P, T	BG, TH	71.00
22	F	4.00	Full	MCA infarction & WMDI	L>R	-	CR	N/A

M, male; F, female; R, right; L, left; F, frontal; P, parietal; T, temporal; O, occipital; BG, basal ganglia; WMDI, white matter damage of immaturity; IVH, ; MCA, middle cerebral artery; HIE, hypoxic ischemic encephalopathy; -, no finding in this field, TH, thalamus; CR, corona radiata; PO, pons; FSIQ, full-scale intelligence quotient; N/A, not applicable.

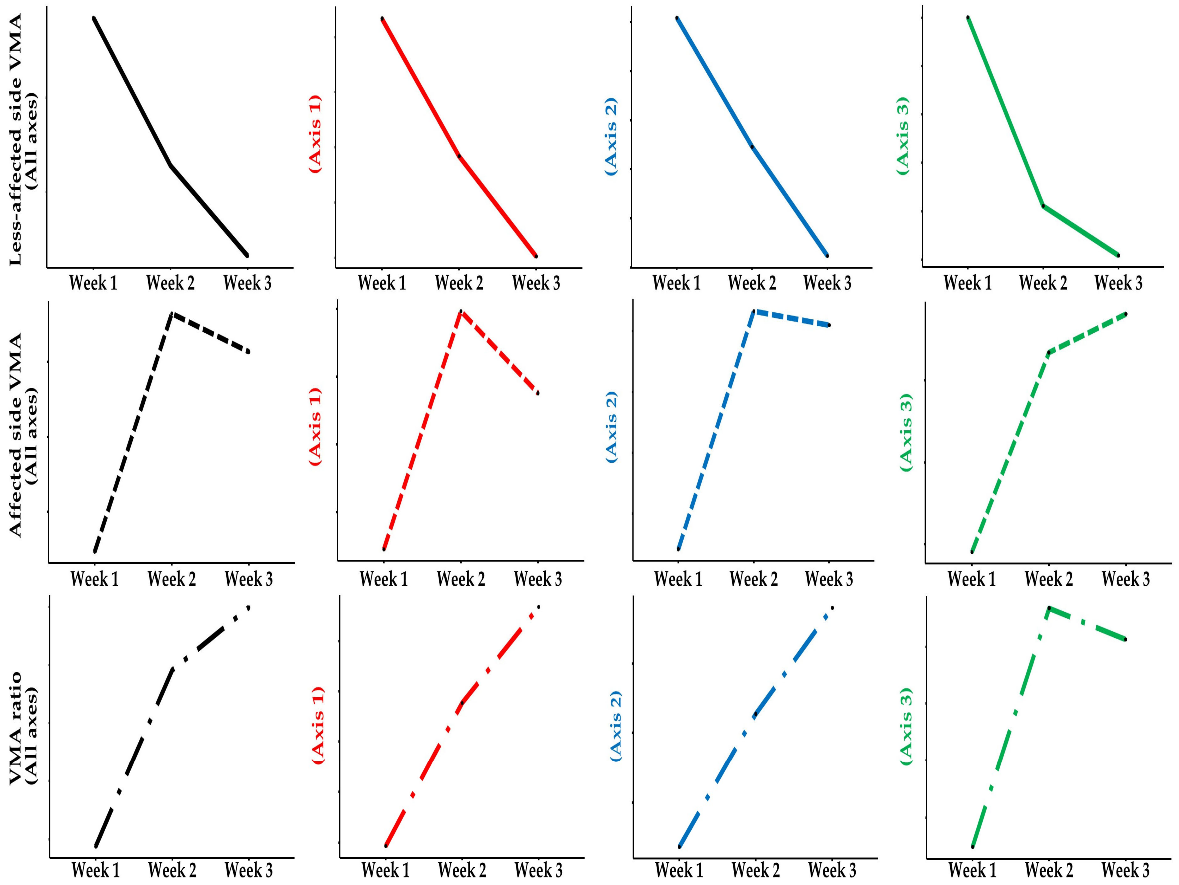

Supplementary Figure S1. Weekly Trends in Actigraphy Outcomes During CIMT. The figure delineates the trajectory of actigraphy readings across three weeks of CIMT. Divided into 12-line plots, it contrasts activity trends between the less-affected (solid line) and affected sides (dashed line), and also illustrates their combined VMA Ratio (mixed solid and dashed line). The vertical breakdown of plots rep-resents different dimensions: all axes (black), Axis 1 (red), Axis 2 (blue), and Axis 3 (green).
